# Checkpoint Kinase ATR Phosphorylates Cdt2, a Substrate Receptor of CRL4 Ubiquitin Ligase, and Promotes the Degradation of Cdt1 following UV Irradiation

**DOI:** 10.1371/journal.pone.0046480

**Published:** 2012-09-28

**Authors:** Hiroki Sakaguchi, Toshihiro Takami, Yoshinori Yasutani, Takeshi Maeda, Masayuki Morino, Takashi Ishii, Yasushi Shiomi, Hideo Nishitani

**Affiliations:** Graduate School of Life Science, University of Hyogo, Kamigori, Ako-gun, Hyogo, Japan; University College London, United Kingdom

## Abstract

The DNA replication-licensing factor Cdt1 is present during the G1 phase of the cell cycle. When cells initiate S phase or are UV-irradiated, Cdt1 is recruited to chromatin-bound PCNA and ubiquitinated by CRL4^Cdt2^ for degradation. In both situations, the substrate-recognizing subunit Cdt2 is detected as a highly phosphorylated form. Here, we show that both caffeine-sensitive kinase and MAP kinases are responsible for Cdt2 phosphorylation following UV irradiation. We found that Cdt1 degradation was attenuated in the presence of caffeine. This attenuation was also observed in cells depleted of ATR, but not ATM. Following UV irradiation, Cdt2 was phosphorylated at the S/TQ sites. ATR phosphorylated Cdt2 *in vitro*, mostly in the C-terminal region. Cdt1 degradation was also induced by DNA damaging chemicals such as methyl methanesulfonate (MMS) or zeocin, depending on PCNA and CRL4-Cdt2, though it was less caffeine-sensitive. These findings suggest that ATR, activated after DNA damage, phosphorylates Cdt2 and promotes the rapid degradation of Cdt1 after UV irradiation in the G1 phase of the cell cycle.

## Introduction

Ordered progression of the cell cycle is controlled by the periodic activation of cyclin-dependent kinases (CDKs) [1]. Ubiquitin-mediated proteolysis by the proteasome is also essential for proper progression of cell-cycle events [2], [3]. There are two major E3 ubiquitin ligases operating during the cell cycle. One is anaphase-promoting complex/cyclosome (APC/C), which functions from the M phase to the G1 phase [3]. The other is SCF, comprising Skp1, Cullin 1 (Cul 1), and one of the F-box family proteins. SCF mainly functions from the late G1 to S and G2/M phases to control CDK activity [4]. SCF is also known as CRL1, Cullin-ring ligase 1 [5], [6]. Cul1 is a scaffold protein that assembles the complex, F-box protein is a substrate-recognizing subunit, and Skp1 is an adaptor that connects Cul1 and the F-box protein.

Recent data demonstrated that ubiquitin-mediated proteolysis is essential for ensuring that DNA replication occurs only once per cell cycle [7]. Replication origins are “licensed” for replication by forming a pre-replicative complex in the late M phase or G1 phase. Licensing is performed by loading the MCM2–7 complex with the aid of Cdc6 and Cdt1 onto the sites bound by the origin-recognition complex (ORC) [8], [9], [10]. Among these factors, Cdt1 levels are strictly regulated in mammalian cells [11]. Cdt1 accumulates during the G1 phase, but is degraded and maintained at a low level once DNA replication is initiated to prevent re-replication of chromosomes. Mammalian Cdt1 was first shown to be proteolysed by a CRL1^Skp2^-mediated pathway [12], [13]. Cullin4 (Cul4) containing E3, Cul4-DDB1-Cdt2 (also known as CRL4^Cdt2^) was subsequently shown to be central to Cdt1 proteolysis in yeast to mammalian cells [14], [15], [16], [17], [18], [19]. Cdt2 is a WD40 repeat-containing protein isolated as a DDB1 binding protein and is required for Cdt1 proteolysis. Importantly, Cdt1 binds to PCNA through a PIP box located at the N-terminus, which is essential for CRL4^Cdt2^-mediated ubiquitination. PCNA connects Cdt1 and CRL4^Cdt2^ on the chromatin. Interestingly, Cdt1 is degraded after DNA damage, such as UV irradiation, by the same PCNA-mediated CRL4^Cdt2^ pathway [14], [15], [16], [17], [18], [19], [20], [21]. Following local UV or laser irradiation, both Cdt1 and CRL4^Cdt2^ are rapidly recruited to the damaged sites dependent on the chromatin association of PCNA [22], [23]. Detailed analyses using *Xenopus* egg extracts demonstrated that either the initiation of replication or incubation with damage-containing DNA triggers chromatin loading of PCNA, the association of Cdt1 with PCNA through its PIP box, and the recruitment of Cdt2 [14], [24]. PCNA loader proteins also regulate Cdt1 degradation. The largest loader protein, RFC1, is required for Cdt1 degradation following UV irradiation, while another protein, Ctf18, is required during the S phase [25]. Other proteins downregulated by the CRL4^Cdt2^ pathway include p21, Xic1, and Set8 in vertebrates [26], [27], [28], [29], [30], [31], [32], [33], [34]. These proteins share conserved amino acids within and downstream of the PIP-box, creating a specialized degron for the CRL4^Cdt2^ pathway [24], [35].

UV irradiation induces helix-distorting DNA damage such as cyclobutane pyrimidine dimers and 6-4 photoproducts, which trigger several signaling cascades to provoke a cellular response that includes a DNA damage-induced checkpoint response. A replication block due to lesions during the S phase triggers the efficient activation of ATR [36]. In the G1 and G0 phases, checkpoint signaling is also activated during the process of nucleotide excision repair (NER), though the level of activation is much lower than that in the S phase [37]. NER is a versatile system for repairing UV-induced DNA lesions. More than 20 proteins, including the 7 xeroderma pigmentosum-related proteins, are involved in NER dual incision, which removes damage-containing oligonucleotides. The resulting gap has a 3′-OH terminus and a single stranded region that is structurally similar to the replication intermediates. Such intermediates appear to be responsible for the ATR-induced phosphorylation of Chk1, p53, and H2AX [37], [38]. PCNA is also loaded on such a 3′-OH terminus-containing intermediate by the aid of RFC1-RFC for the repair synthesis, which is important for CRL4^Cdt2^-mediated degradation of Cdt1 [25], [39]. Besides DNA damage-mediated checkpoint signaling, UV irradiation activates various MAP kinases, such as JNK, p38, and ERK [40].

Cdt2 contains seven WD40 repeats in the N-terminal half part, which is conserved from yeast to mammals and is thought to form a substrate-recognizing propeller structure. In contrast to yeast, Cdt2 of higher eukaryotic cells has a long C-terminal region. We previously demonstrated that Cdt2 was highly phosphorylated following UV irradiation. Here, we examined whether any kinases regulate Cdt1 degradation following UV irradiation. CRL4-Cdt2 mediated Cdt1 degradation was independent of ATR/ATM [20]. We demonstrate here that Cdt1 degradation was delayed in the absence of ATR. ATR phosphorylated purified Cdt2 protein *in vitro*, and Cdt2 that was recovered after UV irradiation contained phosphorylated S/TQ sites. These findings suggest that the checkpoint response is involved in the rapid degradation of Cdt1 after DNA damage.

## Results

### Phosphorylation of Cdt2 by multiple kinases after UV irradiation

**Figure 1:**
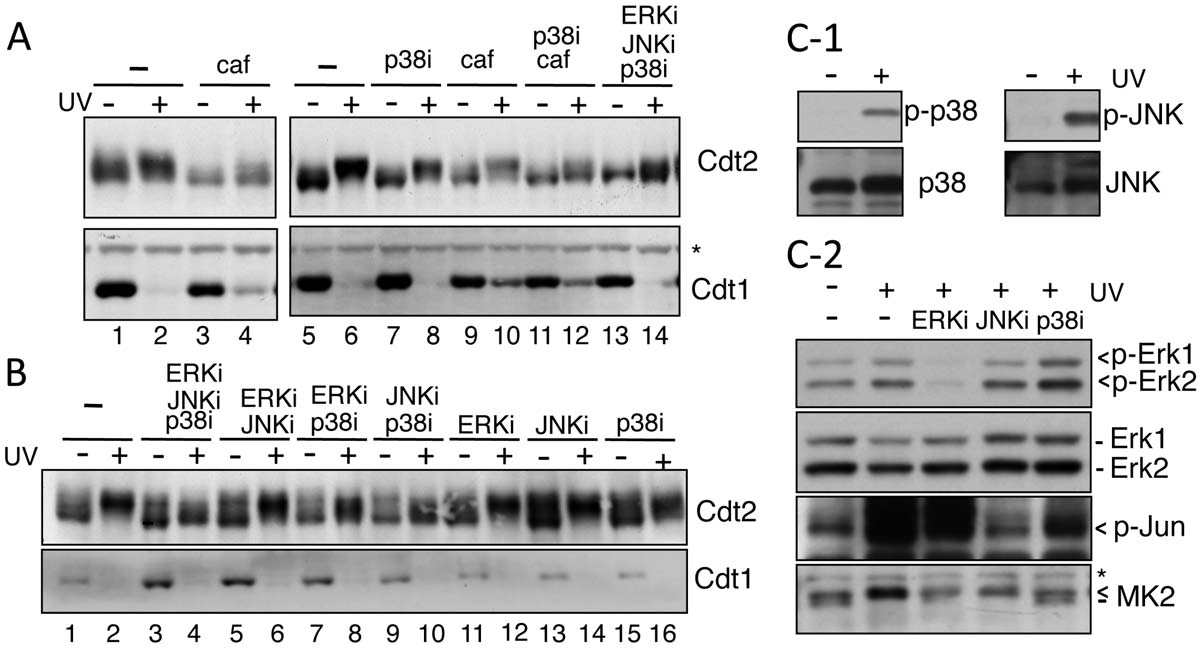
Cdt2 phosphorylation after UV irradiation is caffeine- and MAP kinase-inhibitor sensitive. A. HeLa cells synchronized in the G1 phase were treated with the indicated inhibitors for 2 h and UV-irradiated (+) or not (−). Cell extract was blotted with Cdt1 and Cdt2 antibodies. Inhibitors used were: ERKi, U0126; JNKi, SP600125; p38i, SB202190. The asterisk indicates a non-specific band, and used as a loading control. B. Asynchronously growing HeLa cells were treated with the indicated MAP kinase inhibitors and UV-irradiated (+) or not. One hour later, cells were examined for Cdt2 phosphorylation levels and Cdt1 protein levels. Cdt2 phosphorylation levels were examined using Phos-tag gel. C. Target specificity of inhibitors. Activation of each MAP kinase was examined using specific phosphopeptide antibodies (Erk1 and 2, JNK or p38) before (−) and after (+) UV-irradiation (C-1 and 2). Cells, treated or not with the indicated inhibitors for 2 h, were UV-irradiated or not. One hour later, cell extracts were prepared and their phosphorylation levels of ERK (Erk1 and Erk2), Jun, and MAPKAPK2 (MK2) were examined(C-2). Note that U0126 inhibits the ERK pathway by inhibiting ERK1-activating kinases MEK1 and MEK2. Jun is a JNK substrate and MAPKAPK2 is a p38 substrate. Arrow in MK2 Western blotting indicates the phosphorylated form of MK2. The asterisk indicates a non-specific band.

We previously reported that the phosphorylation levels of Cdt2 protein are low during the G1 phase when Cdt1 is present at high levels, but that as cells enter the S phase or when cells are exposed to UV irradiation, the Cdt2 phosphorylation levels increase and Cdt1 is degraded [22]. Thus, we were interested in how Cdt2 phosphorylation following UV irradiation is linked to the degradation of Cdt1. Many protein kinases, such as ATR/ATM and MAP kinases, are activated following UV irradiation. To investigate the kinase(s) that phosphorylate Cdt2 after UV irradiation, we first treated HeLa cells with caffeine, an ATR/ATM inhibitor. Cells were synchronized in the G1 phase, when Cdt1 was present. Caffeine partially inhibited Cdt2 phosphorylation (Fig. 1A, compare lanes 1 and 2 with lanes 3 and 4). We next examined whether any MAP kinases were involved in Cdt2 phosphorylation. Activation of the MAP kinases ERK, JNK, and p38 was monitored by phosphorylation of each kinase using specific anti-phospho antibodies. All MAP kinases, specially JNK and p38, were activated following UV irradiation (Fig. 1C). Then, we treated the cells with three specific MAP kinase inhibitors for ERK (U0126), JNK (SP600125), and p38 (SB202190) pathways, and examined the Cdt2 phosphorylation levels after UV irradiation. Specific inhibition of each kinase was confirmed by analyzing the phosphorylation levels of relevant substrates; phosphorylation of ERK1 and ERK2 was inhibited by U0126, that of jun by SP600125, and that of MAPKAPK2 by SB202190 (Fig. 1C) [41]. Although incubation with each inhibitor alone had almost no effect on the Cdt2 phosphorylation levels, the p38 inhibitor SB202190 seemed to have a greater effect than the other two, because in combination with ERK or JNK inhibitors, it reduced Cdt2 phosphorylation levels more than double treatment with ERK and JNK inhibitors (Fig. 1B, compare lane 6 with lane 8 or 10). Its inhibitory effect on Cdt2 phosphorylation increased when combined with both the JNK and ERK inhibitors (Fig. 1B, lane 4). Careful examination using a cell culture synchronized in the G1 phase also indicated that Cdt2 phosphorylation levels were reduced in the presence of the p38 inhibitor in combination with caffeine or another MAP kinase inhibitors (Fig. 1A, lane 12 and 14). These findings suggest that Cdt2 was phosphorylated by multiple kinases after UV exposure.

### Caffeine reduced the degradation rate of Cdt1 after UV irradiation

**Figure 2:**
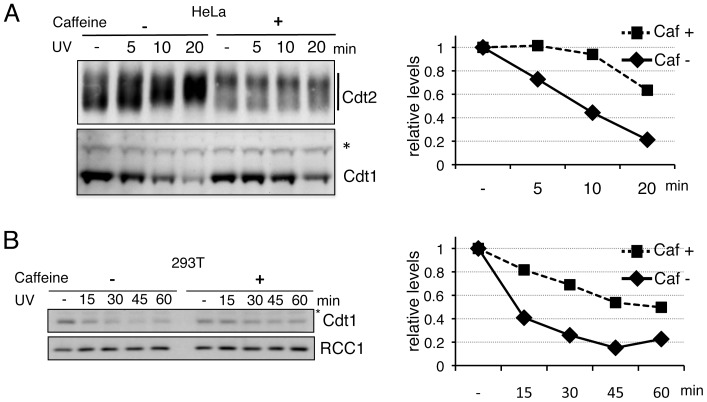
Caffeine inhibits UV-induced rapid degradation of Cdt1. A. Asynchronously growing HeLa cells treated with caffeine (+, 10 mM) or not (−) were UV-irradiated (50 J/m^2^) and collected for Western blotting with Cdt1 and Cdt2. For Cdt2 blotting, samples were run on Phos-tag gels. The asterisk indicates a non-specific band used as a loading control. Cdt1 protein levels are presented as values standardized to 1.0 for UV(−) cells. B. 293T cells were treated as in A, except UV irradiation at 20 J/m^2^, and Cdt1 levels were examined. Cdt1 levels were normalized with RCC1, and are presented as values standardized to 1.0 for UV(−) cells.

Because there was a good correlation between the Cdt2 phosphorylation levels and the degradation of Cdt1 [22], we examined the effect of inhibitors on Cdt1 degradation. Cdt1 was degraded in 1 h in control HeLa cells. In contrast, a substantial amount of Cdt1 remained in the caffeine-treated cells (Fig. 1A, lanes 4, 10, and 12). MAP kinase inhibitors had no inhibitory effect on Cdt1 degradation. To confirm the inhibitory effect of caffeine on Cdt1 degradation, we performed a time-course analysis of Cdt1 degradation. In the absence of caffeine, half of the amount of Cdt1 was degraded in 10 min after UV irradiation at a dose of 50 J/m^2^ and retarded forms of Cdt2 were detected. In contrast, in the presence of caffeine, Cdt1 degradation was delayed and the retarded form of Cdt2 was not clearly detected (Fig. 2A). We observed a similar delay of Cdt1 degradation in 293T cells (Fig. 2B).

### ATR was required for rapid Cdt1 degradation

**Figure 3:**
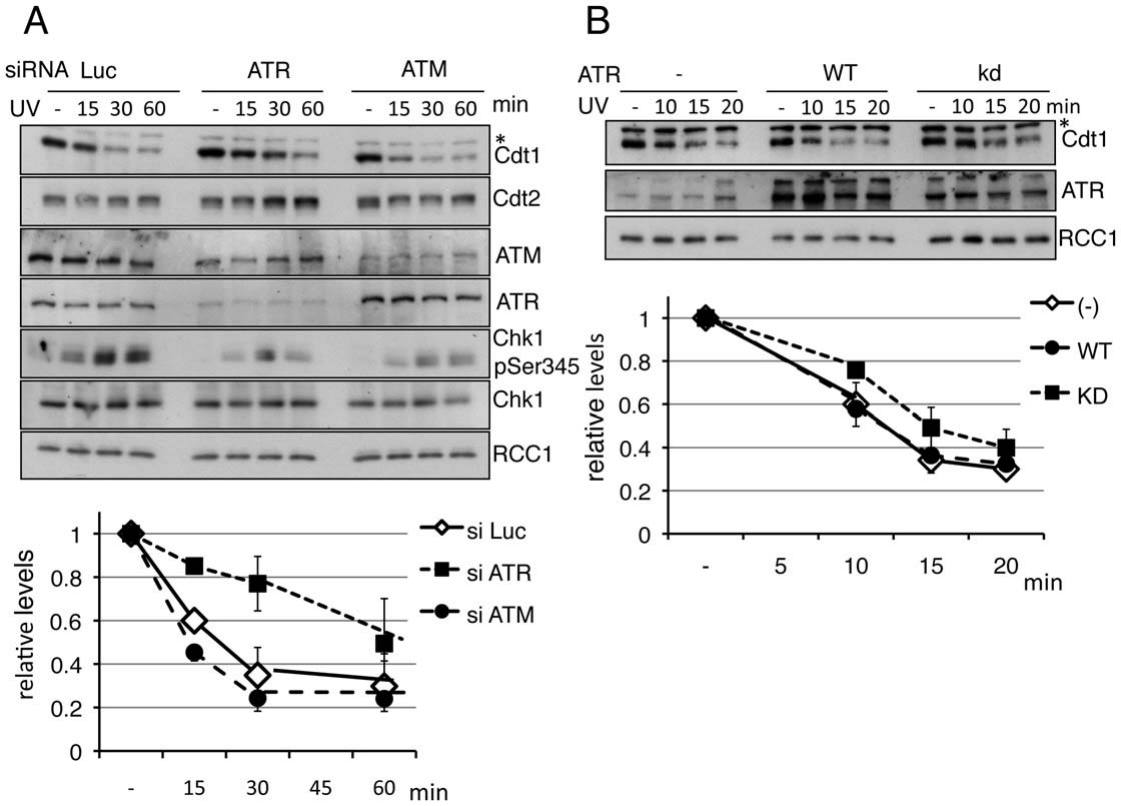
ATR promotes rapid degradation of Cdt1 after UV irradiation. A. 293T cells were transfected with siRNAs for luciferase (Luc, as a control), ATR or ATM. Two days later, cells were UV-irradiated (20 J/m^2^) and collected at the indicated time points for Western blotting. Cdt1 levels were normalized with RCC1, and are presented as values standardized to 1.0 for UV(−) cells. B. 293T cells were transfected with empty vector, ATR (wild-type, wt), or ATR (kinase-dead, kd) expression vector. Two days later, cells were UV-irradiated (20 J/m^2^) and collected at the indicated time points for Western blotting. Cdt1 levels were normalized with RCC1, and are presented as values standardized to 1.0 for UV(−) cells.

The above results suggested that ATR or ATM was involved in the rapid degradation of Cdt1 after UV irradiation. To confirm this, we depleted ATR and ATM from 293T cells using small interfering RNA (siRNA) and examined the rate of Cdt1 degradation. In control siRNA-treated cells, Cdt1 was degraded to 50% within 30 min. In ATR-depleted cells, Chk1 phosphorylation levels were reduced and Cdt1 degradation was delayed similar to that in caffeine-treated cells (Fig. 3A). Such a delay was not observed in ATM-depleted cells, demonstrating that Cdt1 degradation following UV irradiation was promoted by ATR. To confirm the role of ATR on Cdt1 degradation, we overexpressed wild-type or kinase-dead ATR in 293T cells and examined the degradation kinetics. The rate of Cdt1 degradation was not changed after transfection with wild-type ATR, probably because the endogenous levels of ATR were sufficient for rapid degradation. In contrast, degradation was delayed in cells transfected with kinase-dead ATR, suggesting that this form of ATR had a dominant negative effect on Cdt1 degradation, when overexpressed (Fig. 3B). These findings imply that ATR kinase is required for the rapid degradation of Cdt1 following UV irradiation.

### Cdt2 was phosphorylated by ATR in vitro

**Figure 4:**
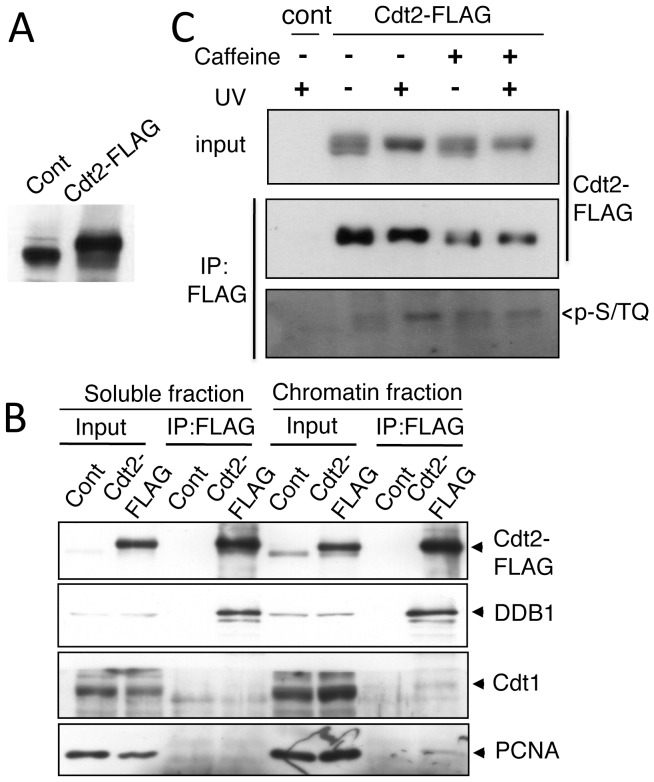
Cdt2 was phosphorylated at S/TQ sites following UV irradiation. A. HEK293 cells (cont) and HEK293 cell line stably expressing Cdt2-3xFLAG (Cdt2-FLAG) were blotted with FLAG antibodies. B. Cdt2-3xFLAG isolated from chromatin-containing fractions of control or Cdt2-3xFLAG expressing cells after UV irradiation was co-purified with DDB1, Cdt1, and PCNA. C. Cells treated with caffeine (+) or not (−) were UV-irradiated at 100 J/m^2^ (+) or not (−), and Cdt2-3xFLAG was immunoprecipitated and immunoblotted with anti p-S/TQ and FLAG antibodies.

Cdt2 has several SQ/TQ motifs that are found in ATM/ATR substrates. The above results suggested that checkpoint kinase ATR was activated to degrade Cdt1 following UV irradiation, so we next examined if ATR was directly involved in Cdt2 phosphorylation. We isolated a HEK293 cell line that stably expresses Cdt2-FLAG with about five times more Cdt2 protein than that in control cells (Fig. 4A). The tagged Cdt2 was functional, because it formed a complex with CRL4 components, Cul4 and DDB1, and the Cdt2-FLAG isolated after UV irradiation from the chromatin-containing fraction was co-purified with PCNA and Cdt1 (Fig. 4B). Using this cell line, Cdt2-FLAG protein was immunoprecipitated with anti-FLAG antibodies after UV irradiation and immunoblotted with an anti-phospho S/TQ specific antibody that recognizes ATR/ATM-phosphorylated sites. The Cdt2-FLAG immunoprecipitated from UV-irradiated cells, but not from non-irradiated cells, was recognized with anti-p-S/TQ antibodies (Fig. 4C). In the presence of caffeine, such an increase in p-S/TQ phosphorylation levels was not observed.

**Figure 5:**
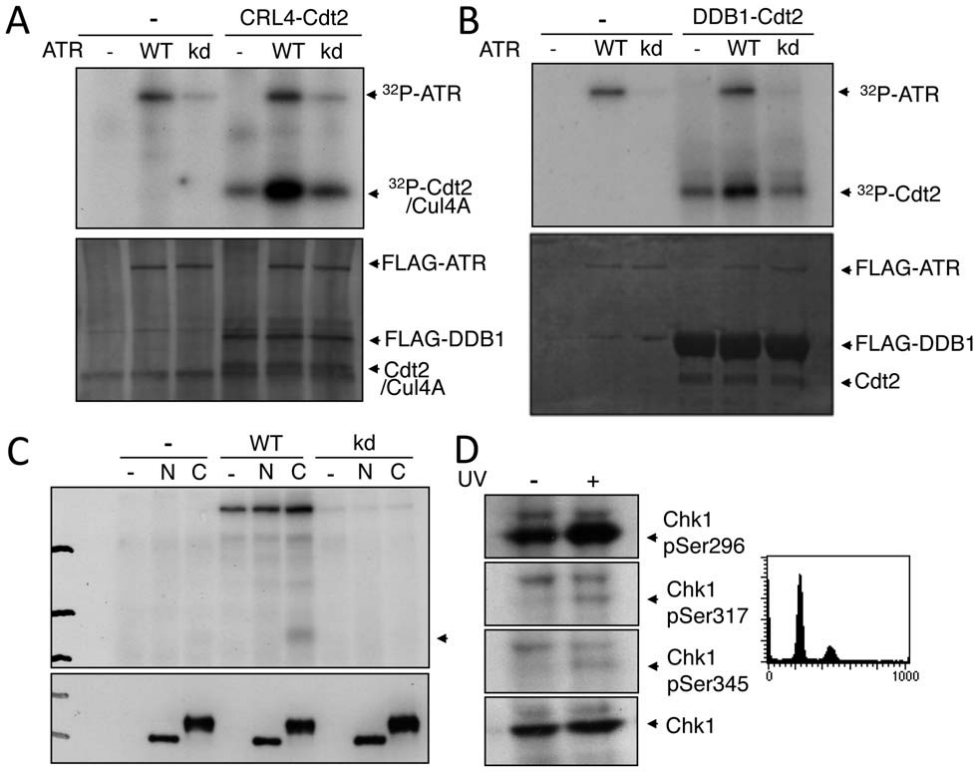
Cdt2 was phosphorylated by ATR. A. For the kinase assay, purified CRL4-Cdt2 complex was incubated in the presence of ^32^P-ATP with anti-FLAG immunoprecipitates from 293T cells transfected with vector (−), or constructs encoding FLAG-tagged wild-type ATR (WT) or kinase-dead ATR (kd). Levels of phosphorylated Cdt2/Cul4A (^32^P) and silver-stained ATR and Cdt2/Cul4A are shown. B. DDB1(FLAG)-Cdt2 complex, expressed with baculo virus system and immunoprecipitated with FLAG antibody, was assayed for phosphorylation as described above. Proteins were stained with Coomassie brilliant blue. C. FLAG-Cdt2(2-400) and FLAG-Cdt2(390-end) were expressed in 293T cells, immuno-purified, and used for the ATR kinase assay as described above. Levels of phosphorylation (top) and proteins (bottom, Western blotting) are shown. D. HEK293 cells synchronized in G1 phase were UV-irradiated (+) or not (−) and Western blotted with the indicated antibodies.

We then investigated whether Cdt2 was a direct substrate for ATR using an ATR immune complex kinase assay. For this assay, we first used a purified CRL4-Cdt2 complex as the substrate [30]. The incorporation of ^32^P was evident at the Cdt2/Cul4A position, but not at DDB1, when the complex was incubated with wild-type ATR (Fig. 5A). In contrast, the phosphorylation levels were reduced to control levels when the kinase-dead mutant of ATR(kd) was used. Since Cdt2 and HA-tagged Cul4A of our CRL4-Cdt2 complex migrated to the same position on SDS-PAGE, it was possible that either Cdt2 or Cul4A or both proteins were phosphorylated by ATR. To confirm that Cdt2 was a target of ATR, we then purified Cdt2 from cells as a FLAG-DDB1/Cdt2 complex and used it for a kinase assay. Cdt2 was efficiently phosphorylated by wild-type ATR (wt) but not by a kinase-inactive ATR (kd), demonstrating that Cdt2 was a direct target of ATR (Fig. 5B). The phosphorylation assay was carried out using the N-terminal and C-terminal half of Cdt2. The C-terminal part of Cdt2 was phosphorylated. The phosphorylation level was lower than that in the assay using purified Cdt2, probably due to the low amounts of Cdt2 in the immunoprecipitates used for the assay (Fig. 5C). UV-induced degradation of Cdt1 occurs in the G1 phase when Cdt1 is present, thus we verified the activation of ATR during this phase of the cell cycle by demonstrating the appearance of Chk1 phosphorylated at specific sites (Fig. 5D), though their levels were low, which was consistent with a previous report [36].

### Cdt1 degradation following treatment with DNA damaging drugs

**Figure 6:**
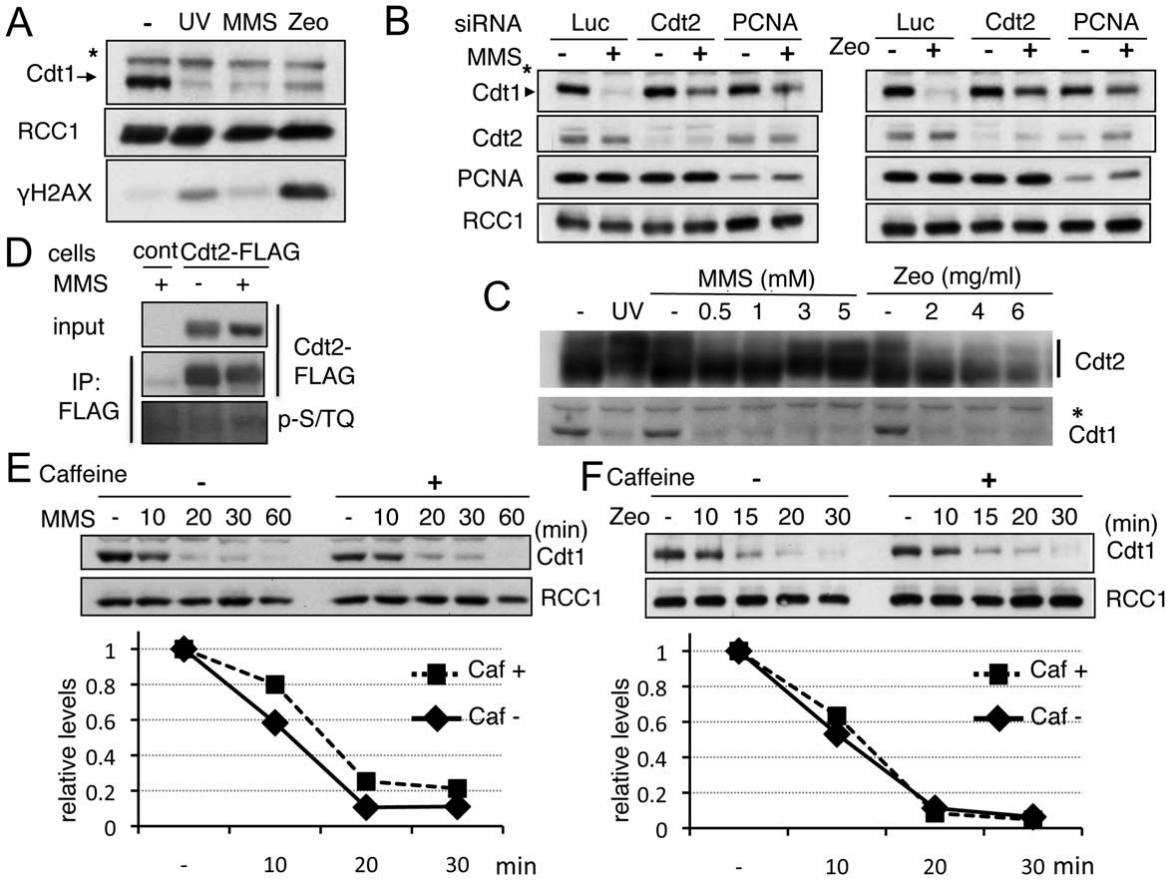
DNA damaging chemical-induced Cdt1 degradation. A and B. DNA damage-inducing reagent MMS or zeocin-induced PCNA-CRL4Cdt2 mediated degradation of Cdt1. 293T cells were treated with MMS (0.5 mM) or zeocin (2 mg/ml) for 1 h, and Cdt1 levels and γH2AX levels were examined (A). Cells were transfected with siRNAs for luciferase (Luc), Cdt2 or PCNA, and then treated with MMS or zeocin. Protein levels of indicated proteins were examined. C. 293T cells were treated with the indicated doses of MMS or zeocin, and collected 1 h later, and Cdt2 and Cdt1 were examined. D. HEK293 (cont) or Cdt2-3xFLAG expressing cells were treated with MMS (3 mM). Cdt2-3xFLAG was immunoprecipitated, and Western blotting was performed with anti p-S/TQ and FLAG antibodies. E and F. 293T cells were incubated with caffeine (10 mM) for 2 h, and then treated with MMS (0.5 mM) or zeocin (2 mg/ml). Cells were collected at the indicated times, and Cdt1 levels were examined. Cdt1 levels were normalized with RCC1, and are presented as values standardized to 1.0 for untreated cells (−).

The effect of different DNA damaging chemicals on Cdt1 degradation in mammalian cells is not clear, thus we analyzed Cdt1 degradation after treatment with methyl methanesulfonate (MMS), an alkylating agent, and zeocin, a gamma radiation-mimicking reagent that creates strand breaks, including double-strand breaks. Both of these reagents induced Cdt1 degradation, depending on the PCNA-CRL4^Cdt2^ pathway, because depletion of either Cdt2 or PCNA inhibited Cdt1 degradation (Fig. 6A and 6B). We then examined if these reagents induced Cdt2 phosphorylation. When the cells were treated with MMS, similar slowly migrating bands of Cdt2 were observed in a dose-dependent manner (Fig. 6C). In zeocin-treated cells, Cdt1 was degraded, however, we could not detect any increase of slowly migrating forms of Cdt2. We also examined the phosphorylation levels at S/TQ sites and the effect of caffeine after MMS- or zeocin-treatment. Cdt2 was phosphorylated at the S/TQ sites after MMS-treatment, but at a lower level than that observed after UV irradiation (Fig. 6D), and the effect of caffeine on degradation rate was also lower (Fig. 6E). Phosphorylation at S/TQ sites was not detected after zeocin treatment (data not shown), consistent with the observation that there was no change in Cdt2 phosphorylation levels after zeocin treatment. In addition, Cdt1 was degraded at the same rate in the presence of caffeine (Fig. 6F). Although Cdt1 was degraded following treatment with the DNA-damaging chemicals MMS and zeocin, their dependence on ATR appears to be lower than that after UV irradiation.

## Discussion

Licensing factor Cdt1 is rapidly degraded within minutes following UV irradiation. Here, we demonstrated that checkpoint kinase ATR is required for this rapid degradation of Cdt1. The phosphorylation levels of Cdt2, a substrate receptor of CRL4 ubiquitin ligase, is normally low during the G1 phase. Its levels increase not only when cells enter the S phase, but also when cells are irradiated with UV, and Cdt1 is degraded in both cases. The present findings suggest that both MAP kinases and ATR are involved in Cdt2 phosphorylation following UV irradiation. Importantly, ATR, but not ATM or MAP kinases, is required for the rapid degradation of Cdt1. An *in vitro* kinase assay demonstrated that Cdt2 protein was phosphorylated by ATR, and Cdt2 isolated following UV irradiation contained phosphorylated S/TQ sites. Human Cdt2 has nine SQ sites and quantitative phosphoproteomic analyses reveal its phosphorylation at several of these sites [42], [43].

ATR activation following UV irradiation was reported in the S phase [36]. UV-induced DNA damage blocks DNA replication fork progression and leads to the recruitment of ATR and its activation [36]. ATR is also activated in G1 phase during the process of NER, when the UV-induced photoproducts are removed and a single-stranded region is formed [37], [38], [44]. ATR activation is enhanced by the action of Exo1, which produces larger ssDNA gaps [45], [46]. Although Cdt1 degradation occurs in the absence of ATR and ATM, as previously reported [20], the present findings suggest that ATR phosphorylation of Cdt2 promotes Cdt1 degradation. The single-stranded DNA-gap produced during NER contains a 3′-OH terminus and 5′ DNA junction. PCNA is loaded at the 3′-OH terminus and recruits both Cdt1 and CRL4^Cdt2^ [25], [39]. On the other hand, the checkpoint clamp 9-1-1 may be loaded at the 5′ junction of the gap, because it is preferentially loaded at the 5′ DNA junction [47], [48]. The loaded 9-1-1 will activate ATR to phosphorylate Cdt2. Consistent with this, Rad9 protein foci are detected after UV irradiation [49]. Rapid proteolysis of Cdt1 might improve the accessibility of repair enzymes such as DNA polymerases to the chromatin-bound PCNA. Conversely, it is possible that the first recruitment of Cdt1 to the PCNA-loaded sites transiently blocks the repair synthesis and the resulting ssDNA region is then required for efficient checkpoint activation at a very early stage of DNA damage checkpoint signaling. Once ATR is activated, it will enhance Cdt1 degradation for efficient repair.

How does ATR-mediated phosphorylation of Cdt2 promote Cdt1 degradation? In contrast to fission yeast Cdt2, higher eukaryotes have Cdt2 with an extended C-terminal region. As indicated by quantitative phosphoproteomic analyses, phosphorylation is observed mainly in the C-terminal part. Our *in vitro* kinase assay also indicated that ATR phosphorylated mostly the C-terminal part of Cdt2. Phosphorylation may modulate the affinity between PCNA, Cdt1, and CRL4^Cdt2^ in conjunction with DNA on the chromatin, and increase the activity of CRL4^Cdt2^. We examined whether the chromatin association of Cdt2 was affected in the ATR-depleted cells, but observed no change in the affinity to chromatin (data not shown). Recently, many factors necessary for Cdt1 degradation after UV irradiation were reported through genome-wide screening, such as NER factors, COP9/signalsome, and p97 AAA+-ATPase [39]. It is proposed that p97 is involved in Cdt1 degradation by facilitating the release of ubiquitinated Cdt1 from PCNA on chromatin. ATR phosphorylation of Cdt2 may also facilitate such an event.

DNA damaging chemicals, MMS and zeocin, also induced Cdt1 degradation. We observed that caffeine reduced the rate of Cdt1 degradation and that Cdt2 was phosphorylated at S/TQ sites following MMS treatment, suggesting that ATR also promotes Cdt1 degradation in this case. However, the effect of caffeine on Cdt1 degradation and the levels of p-S/TQ was lower than those observed after UV irradiation. Following zeocin-treatment, we detected no p-S/TQ phosphorylation on Cdt2 (data not shown) and caffeine had almost no effect on the rate of Cdt1 degradation (Fig. 6F). These results suggest that the levels of ATR activation and thus their effect on Cdt1 degradation are dependent on the relevant repair pathways provoked by different types of DNA damage, such as that induced by UV, MMS or zeocin, in the G1 phase. An alkylating agent such as MMS causes base methylation and subsequent destabilization of glycosidic bonds spontaneously or with the assistance of DNA N-glycosylases, facilitating the production of apurinic/apyrimidinic sites. Such apurinic/apyrimidinic-sites are repaired by base excision repair (BER) [50], [51]. BER is initiated by apurinic/apyrimidinic endonuclease 1, catalyzing the incision of the damaged strand [52]. During a long-patch BER, PCNA, DNA polymerases, and ligase participate in the repair process. PCNA loaded at the nicked site could be used for Cdt1 ubiquitination. Because only 2 to 13 nucleotides are normally replaced during a long-patch BER, ATR activation levels would be low compared with those during NER. This might explain why p-S/TQ levels of Cdt2 were lower after MMS treatment than following UV irradiation. Zeocin causes DNA strand breaks. Double strand breaks in G1 phase are repaired predominantly through non-homologous end-joining. In this case, the levels of ATR activation may be much lower, and even if ATM is activated, its contribution to Cdt1 degradation might be low, as caffeine had almost no effect on Cdt1 degradation (Fig. 6F).

Many anticancer drugs cause DNA damage and induce Cdt1 degradation. A recent report demonstrated that anticancer reagents induced Cdt1 degradation, but the levels of response appeared to be cell specific [53]. In addition, normal cells and cancer cells respond differently to a DNA-replication licensing block [54]. In a normal cell cycle, DNA replication licensing is established at the end of mitosis or in the early G1 phase. Thus, mitosis-specific DNA damage-inducing reagents would be useful chemotherapeutic drugs for cancer cell treatment by degrading Cdt1 and thus inhibiting replication licensing. In addition, ATR is not essential for Cdt1 degradation, but it promotes Cdt1 degradation as demonstrated here. Thus, reagents that will activate ATR are also potential targets to induce the rapid degradation of Cdt1.

## Materials and Methods

### Cell culture

HeLa, HEK293 and HEK293 cells stably expressing Cdt2-FLAG, and HEK293T cells were cultured in Dulbecco's modified Eagle's medium with 10% fetal bovine serum and 5% CO_2_. To synchronize cells in the G1 phase, cells blocked in the early S phase using the thymidine and aphidicolin block method were released for 16 h or nocodazole-arrested cells were released for 3 h. Caffeine was used at 10 mM. MAP kinase inhibitors were used at the following concentrations: ERK inhibitor U0126 (Calbiochem) at 10 µM, JNK inhibitor II (SP600125, Calbiochem) at 30 µM and p38 inhibitor SB202190 (Calbiochem) at 20 µM. Proteasome inhibitor MG132 was used at 25 µM. MMS was used at 0.5 to 5 mM. Zeocin was used at 2–6 mg/ml. UV-C (254 nm) irradiation of whole cells in dishes was performed at 20 to 100 J/m^2^ using a UV cross-linker (FS-800, Funakoshi). To analyze the DNA content, flow cytometry was performed as described previously [15].

### Antibodies and Western blotting

For Western blotting, whole cell lysates were prepared by lysing cell pellets directly in SDS-PAGE buffer. The following primary antibodies were used: Cdt1 (rabbit, [11]); Cdt2 (rabbit, [30]); ATR (Ab-2, Oncogene); ATM (GeneTex); p-S/TQ, Chk1, Chk1 pSer296, Chk1pSer317, Chk1pSer345, ERK, JNK, p38, phospho-jun, and MAPKAPk2 (Cell Signaling); FLAG (F3165, F7425, Sigma-Aldrich); PCNA (PC10, Santa Cruz Biotechnology); and RCC1 [55]. To separate phosphorylated proteins on SDS-PAGE, Phos-tag was purchased from the NARD Institute (AAL-107), and used according to the manufacturer's instructions. Protein levels were analyzed using ImageJ software.

### Immunoprecipitation from Cdt2-FLAG expressing cells

Control or Cdt2-FLAG expressing HEK293 cells that were UV-irradiated or not UV-irradiated were lysed using 0.1% Triton X-100 mCSK buffer: 10 mM Pipes, pH 7.9, 100 mM NaCl, 300 mM sucrose, 0.1(v/v)% Triton X-100, 1 mM phenylmethylsulfonyl fluoride, 10 mM β-glycerophosphate, 1 mM Na_3_VO_4_, and 10 mM NaF. After centrifugation (15,000 rpm for 15 min at 4°C), the supernatants were mixed with anti-FLAG antibody-conjugated magnetic beads (M8823, Sigma-Aldrich) to obtain immunoprecipitates from the supernatants. The precipitates were resuspended in 0.1% Triton X-100 mCSK buffer and sonicated. After centrifugation (30,000 rpm for 20 min at 4°C), the supernatants were used as the chromatin-bound fraction for immunoprecipitation with anti-FLAG antibody-conjugated magnetic beads. Protein expression of FLAG-DDB1 and Cdt2 in Sf21 cells with baculo virus system (pBacPAK6, Clontech), and purification with anti-FLAG column (anti- FLAG M2-agarose, Sigma-Aldrich) was as described [30].

### RNAi knockdown experiments

The double-stranded RNAs were transfected at 100 µM using Oligofectamine (Invitrogen) or HiPerFect (Qiagen). Twenty-four hours after the first transfection, the second transfection was performed and cells were cultured for 2 d. The following siRNAs were made by Dharmacon: PCNA; CGGUGACACUCAGUAUGUC, Cdt2; CCAGGAGGUGAUAAACUUU, ATR; CCUCCGUGAUGUUGCUUGA, ATM; GCGCCUGAUUCGAGAUCCU. The siRNA for siLuc, known as GL2, was used as a control siRNA.

### ATR kinase assay

The assay was performed as described previously [56]. 293T cells were transfected with empty vector pcDNA3.1 or constructs expressing FLAG-wild type ATR or kinase inactive ATR. Two days after transfection, cell lysates were prepared and immunoprecipitated with anti-FLAG antibody-beads. The immuno-complexes were incubated with purified proteins. The following proteins were used: CRL4-Cdt2 complex purified from insect cells [30], FLAG-tagged DDB1-Cdt2 infected into insect cells, isolated with anti-FLAG beads and eluted with FLAG peptides, and FLAG-Cdt2(2-400) or FLAG-Cdt2(390-end) transfected into 293T cells and isolated with anti-FLAG beads.
